# The Operative Role of Artificial Intelligence in Vascular Surgery: A Systematic Review of Literature

**DOI:** 10.7759/cureus.95515

**Published:** 2025-10-27

**Authors:** Naomi Abara, Abdulazeez Mustapha, Revanth Kalichetty, Mohamed Elsaigh

**Affiliations:** 1 Vascular Surgery, Royal Cornwall Hospitals NHS Trust, Truro, GBR; 2 Paediatric Surgery, Birmingham Children's Hospital, Birmingham, GBR; 3 Urology, Royal Cornwall Hospitals NHS Trust, Truro, GBR; 4 General Surgery, Royal Cornwall Hospitals NHS Trust, Truro, GBR

**Keywords:** artificial intelligence, deep learning, machine learning, operative, vascular surgery

## Abstract

This study aims to systematically review existing literature on the impact of artificial intelligence (AI) on operative workflow and safety in vascular surgery. This systematic review was reported in accordance with the Preferred Reporting Items for Systematic Reviews and Meta-Analyses (PRISMA) 2020 guidelines and registered with the International Prospective Register of Systematic Reviews (CRD420251004635). A comprehensive literature search was conducted across PubMed, Embase (via Ovid), Scopus, Google Scholar, and Science Direct for studies published between January 2005 and February 2025. The search strategy used for PubMed, Embase (via Ovid), Scopus, and Google Scholar was: ("artificial intelligence" OR "AI" OR "machine learning" OR "deep learning" OR "neural network") AND ("vascular surgery" OR "endovascular" OR "EVAR" OR "TEVAR" OR "aneurysm repair" OR "carotid endarterectomy" OR "arteriovenous fistula" OR "bypass" OR "inferior vena cava") AND ("segmentation" OR "prediction" OR "risk model" OR "imaging" OR "perioperative" OR "intraoperative" OR "operative planning" OR "workflow" OR "patient safety). The strategy for Science Direct was: ("artificial intelligence" OR "machine learning" OR "deep learning") AND ("vascular surgery" OR "endovascular") AND ("operative role" OR "workflow" OR "patient safety"), as limited to eight words.

The initial search identified 817 studies, and after screening, studies that did not meet the criteria were excluded, leaving eight relevant studies. Six were retrospective studies, one prospective study and one hybrid (retrospective-prospective cohort) study. The exclusion criteria included irrelevant titles, duplicate papers, abstracts, themes and non-English papers.

The review demonstrated predominant utilisation of AI in the preoperative phase for risk prediction, decision support, anatomical assessment, and operative planning. Intraoperatively, AI applications encompassed real-time risk updates and intraoperative guidance based on preoperative computed tomography, while postoperative models enhanced surveillance following endovascular procedures. Across the reviewed studies, conventional methods were often outperformed by AI models in predictive accuracy, workflow efficiency and safety.

AI application shows potential in improving operative workflow and safety in vascular surgery through enhanced decision support, risk prediction, and process automation. However the performance should be interpreted cautiously given current evidence limitations. Overall, the evidence remains limited by small, retrospective, and heterogeneous studies with potential bias, highlighting the need for large-scale prospective validation before routine clinical adoption.

## Introduction and background

Artificial intelligence (AI) has experienced exponential growth over the last two decades, increasingly becoming an integral part of daily life, notably in today's fast-paced technological age. The medical field is not isolated from this advancement in precision. There is interest in the integration of artificial intelligence in various medical and surgical specialties. The continued research in the incorporation of heterogeneous medical data into machine learning and deep learning models is anticipated to facilitate translational improvements in clinical care and limit human error [1].

AI is a specialised domain of computer science that designs models and algorithms to execute tasks, emulating human cognitive abilities. This includes, but is not limited to, detection, reasoning, learning, planning, and prediction [2]. Machine learning (ML) is a subset of AI that gives machines the ability to learn and identify patterns in large data sets without explicit programming [3]. Deep learning networks (DLN), a class of ML, utilize multiple layered neural networks modelled after the human brain to process large data and extract higher-level features from raw data [3].

The existing literature on artificial intelligence in vascular surgery focuses broadly on diagnostics, imaging, outcome prediction, and ethical and legal considerations, with a paucity of information on operative applications and safety. Although AI reviews exist in general surgery, orthopaedics, and neurosurgery, there has been no dedicated synthesis of its operative role and safety in vascular surgery. The peculiar procedural challenges in vascular surgery specifically, endovascular techniques, haemodynamic factors, and high-risk patient groups, further highlight the need for a focused review.

Clinical decision making through insights from past data is the essence of evidence-based medicine and is the global standard in patient care [4]. Traditionally, preoperative decision-making in vascular surgery has relied on clinical judgement, manual imaging interpretation, and conventional research/statistical methods [5-7] for risk assessment and outcome prediction as demonstrated in this review. Operatively, and in comparison, to traditional open surgery, modern vascular techniques including endovascular surgery, robotics, and bioengineering, offer markedly improved patient outcomes through reduced invasiveness, greater precision, and faster recovery, although open surgery remains a necessary and effective option for complex cases [8,9].

This review analyses the integration of AI in key aspects of operative workflow - preoperative planning, intraoperative navigation, decision support, and postoperative monitoring - in patients undergoing vascular surgery, comparing AI-based methods with traditional approaches to evaluate their impact on workflow efficiency and safety across surgical and endovascular settings. It also aims to identify research gaps, highlight areas for future investigation, and outline current limitations to clinical adoption. Recent health policies, for example, the European Union (EU) Artificial Intelligence Act which categorises healthcare AI systems as high-risk [10], highlight the need to evaluate operative applications of AI in vascular surgery, within this evolving regulatory and ethical landscape.

## Review

Methods

This systematic review was reported in accordance with the Preferred Reporting Items for Systematic Reviews and Meta-Analyses (PRISMA) 2020 guidelines [11] and registered with the International Prospective Register of Systematic Reviews (CRD420251004635). The completed PRISMA 2020 checklist is provided in Appendix 1.

Search Strategy

A comprehensive literature search was done by two investigators (NA and MA) on 25th February 2025 across PubMed, Embase (via Ovid), Scopus, Google Scholar and Science Direct published between January 2005 and February 2025. The Boolean search strategy used for PubMed, Embase (via Ovid), Scopus, and Google Scholar was: ("artificial intelligence" OR "AI" OR "machine learning" OR "deep learning" OR "neural network") AND ("vascular surgery" OR "endovascular" OR "EVAR" OR "TEVAR" OR "aneurysm repair" OR "carotid endarterectomy" OR "arteriovenous fistula" OR "bypass" OR "inferior vena cava") AND ("segmentation" OR "prediction" OR "risk model" OR "imaging" OR "perioperative" OR "intraoperative" OR "operative planning" OR "workflow" OR "patient safety). The strategy for Science Direct was: ("artificial intelligence" OR "machine learning" OR "deep learning") AND ("vascular surgery" OR "endovascular") AND ("operative role" OR "workflow" OR "patient safety"), as limited to eight words. All relevant studies carried out from January 2005 to February 2025 were candidates for inclusion.

Inclusion Criteria

Only publications in English and studies involving human subjects were considered. All primary studies conducted between January 2005 and February 2025 that appeared in peer-reviewed journals were included. To be included, all original research articles had to demonstrate AI involvement and relevance to vascular surgery. This was determined by meeting at least one of these criteria: the selected study was published in a vascular focussed journal or in the vascular section of a surgical research journal, or it described interventions relevant to vascular diseases and surgery (for example, endovascular aneurysm repair (EVAR), thoracic endovascular aortic repair (TEVAR), angioplasty, abdominal aortic aneurysms, arterial bypass surgery, peripheral arterial disease, carotid stenosis, thrombectomy amongst others).

Exclusion Criteria

Duplicate papers, irrelevant titles, abstracts, themes, grey literature, or studies unavailable in full-text format were excluded. In addition, we also excluded articles not involving AI, audits, case reports, reviews, meta-analyses, commentaries, editorials, guidelines, conference abstracts/proceedings, non-English papers and experimental studies. Grey literature and conference abstracts were excluded to ensure methodological consistency and data reliability, as they are typically non-peer-reviewed and lack detailed reporting.

*Screening and Selection*

**Figure 1:**
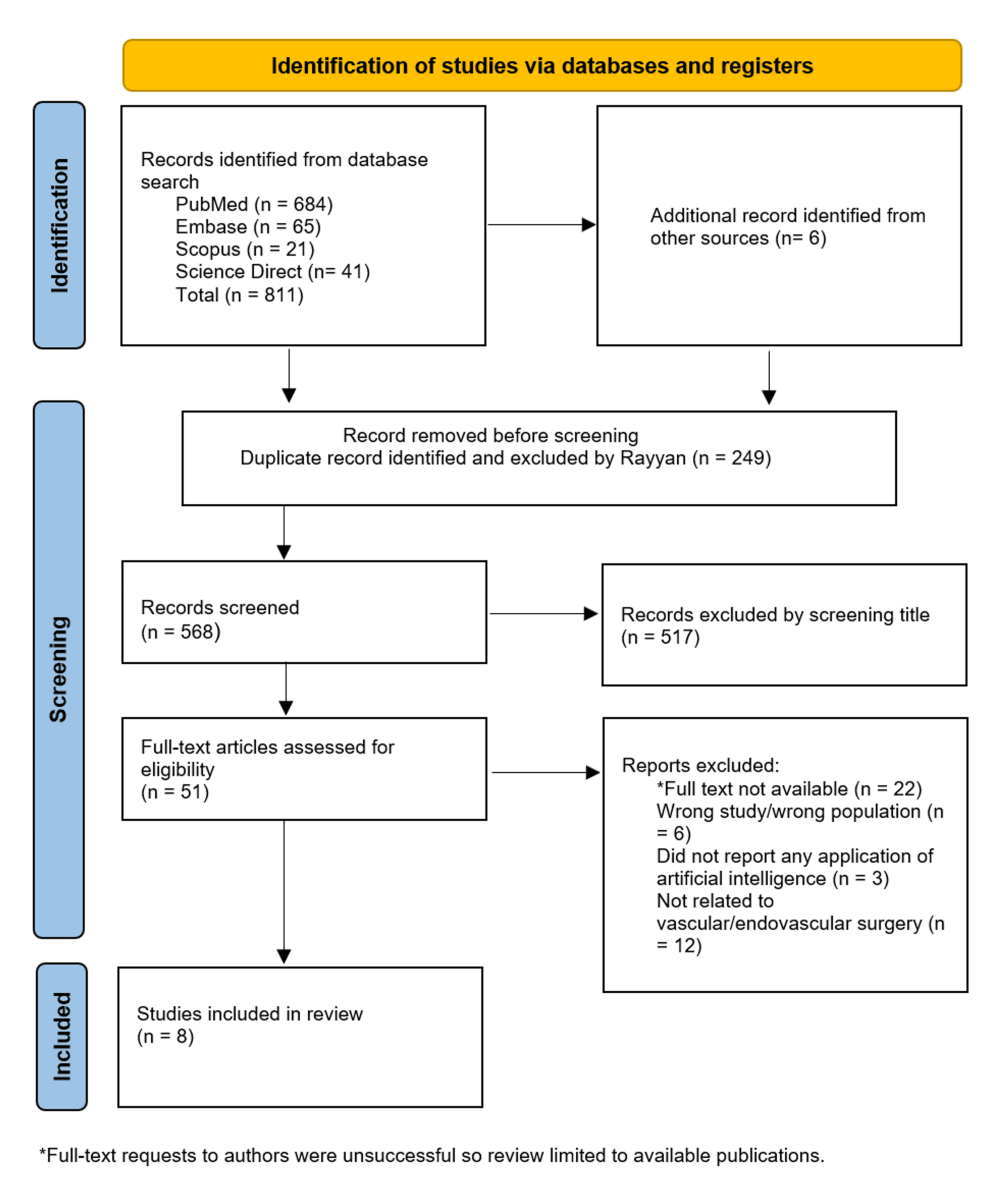
PRISMA 2020 flow diagram for new systematic reviews incorporating database and registers searches only PRISMA: Preferred Reporting Items for Systematic Reviews and Meta-Analyses

A comprehensive search of the database identified 817 studies. As shown in Figure 1, 568 studies remained after removal of the duplicates. Following abstract screening 517 were excluded, leaving 51 papers. A full text review of these 51 papers was done using the inclusion and exclusion guidelines previously mentioned and eight studies fully met the eligibility criteria. The studies spanned a period of 20 years from 2005 to 2025 and included multicentre retrospective, prospective and hybrid studies. Efforts to obtain additional full-text articles from authors were unsuccessful so the review is limited to the available published full-text articles. In accordance with the eligibility criteria and PRISMA guidelines, the included articles were analysed independently by two reviewers (NA and MA) and the proportion of agreement was 94%. Any reviewer discrepancies were resolved through discussions between the two authors until a consensus was reached. Further disagreements were resolved with a third reviewer (RK).

Quality and Risk of Bias Assessment

**Figure 2:**
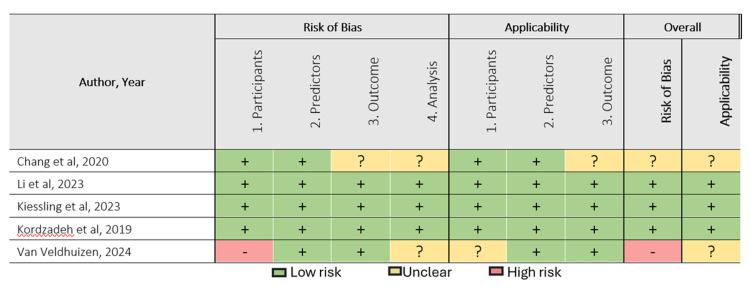
Risk of bias and applicability assessment of included predictive modelling studies using the PROBAST tool PROBAST: Prediction model Risk of Bias Assessment Tool Included predictive modelling studies: Chang et al. [13], Li et al. [14], Kiessling et al. [15], Kordzadeh et al. [16], Van Veldhuizen et al. [17]

**Figure 3:**
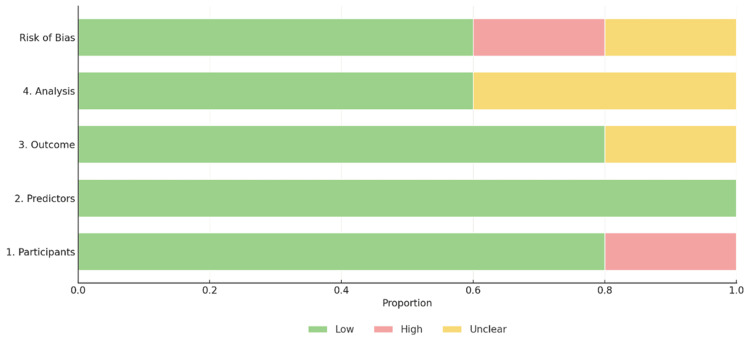
Summary of risk of bias assessment of included predictive modelling studies

**Figure 4:**
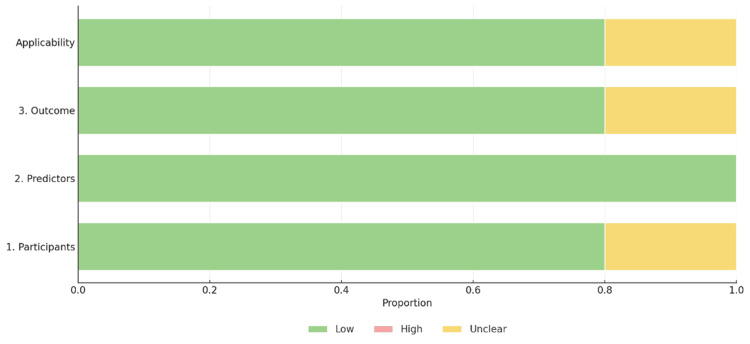
Summary of applicability assessment of included predictive modelling studies

**Figure 5:**
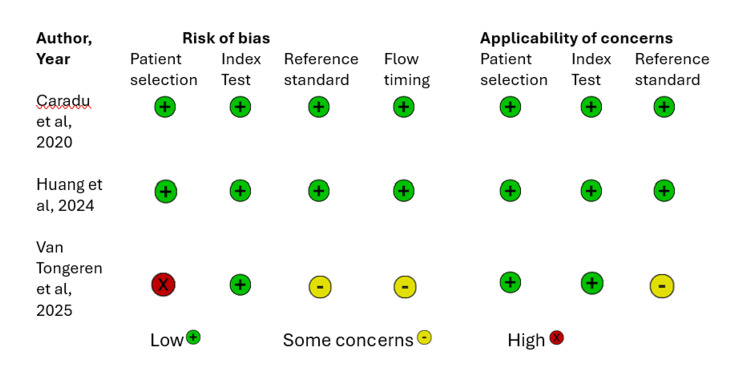
Risk of bias and applicability assessment of included diagnostic imaging studies using the QUADAS-2 tool QUADAS-2: Quality Assessment of Diagnostic Accuracy Studies 2 Included diagnostic imaging studies: Caradu et al. [18], Huang et al. [19], Van Tongeren et al. [20]

**Figure 6:**
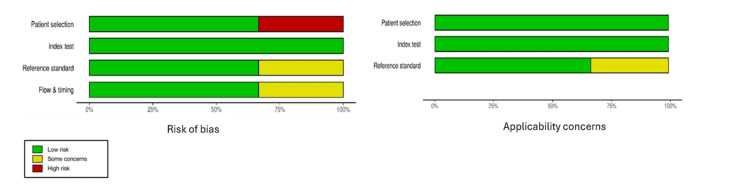
Summary Graph of QUADAS-2 Risk of Bias and Applicability Assessments Across Included Diagnostic Imaging Studies QUADAS-2 -Quality Assessment of Diagnostic Accuracy Studies 2

The quality of the methodological approach in the selected studies was assessed using two tools, the Prediction model Risk of Bias Assessment Tool (PROBAST) and the Quality Assessment of Diagnostic Accuracy Studies 2 (QUADAS-2) tool [2,12], as shown in Figures 2-6. The included studies, as shown in Table 1, encompassed both predictive modelling (five studies) and imaging segmentation or measurement designs (three studies), so methodological quality was appraised using these two tools. PROBAST evaluates the quality, potential bias, and overall applicability of prediction models or algorithms in studies through two components: risk of bias and applicability concerns. Each contains four domains: participants and data sources, predictors, outcome, and analysis [2]. QUADAS-2 is validated to evaluate the quality of diagnostic accuracy studies and assesses risk of bias in four domains: (1) patient selection, (2) index test, (3) reference standard, and (4) flow and timing. Furthermore, the first three domains are evaluated for concerns regarding applicability [12].

**Table 1:** Study design of included articles, AI model applied and clinical domain(s) of impact AAA: Abdominal aortic aneurysm, EVAR: Endovascular aneurysm repair, IVC: Inferior vena cava

Author	Study title	Year of study	Data set	AI tool/software applied	Main clinical domain of impact
Caradu et al. [18]	Fully automatic volume segmentation of infrarenal abdominal aortic aneurysm computed tomography images with deep learning approach versus physician controlled manual segmentation	2020	100 patients preoperative computed tomography angiograms (CTA) images of infrarenal AAA	Imaging model - PRAEVAorta- a software with dedicated algorithms to automatically detect the features of AAA.	Preoperative surgical planning
Chang et al. [13]	Deep learning-based risk model for best management of closed groin incision after vascular surgery.	2020	370 patients who underwent vascular surgery within the study period	Predictive model - Deep learning-based prediction model (DPLM) for patient specific risk of SSI (surgical site infection) after vascular surgery	Preoperative surgical planning
Li et al. [14]	Using machine learning to predict outcomes following carotid endarterectomy (CEA)	2023	166,369 patients who underwent CEA within the study period	Predictive model- XGBoost- a machine learning based prognostic model that guides decision making for patients planned for CEA	Preoperative, intraoperative and postoperative decision support and complication management.
Huang et al. [19]	Virtual-reality robotic-assisted inferior vena cava thrombectomy using virtual vascular endoscopy to identify the inferior vena cava invasion	2024	36 patients with renal tumour and inferior vena cava (IVC) thrombi who underwent robot-assisted inferior vena cava thrombectomy	Imaging model- Virtual vascular endoscopy. Dataset including preoperative computed tomography (CT) were incorporated into deep learning models (DLM) creating realistic image reconstruction that can be viewed virtually to identify IVC invasion.	Preoperative surgical planning
Kiessling et al. [15]	AI outperforms Kaplan-Meier Analyses estimating survival after elective treatment of abdominal aortic aneurysms	2023	7912 patients who underwent elective repair of AAA	Predictive model- Neural multi-task logistic regression (N-MTLR), a machine learning model that captures complex associations in patients’ data and estimates patient specific survival post open surgical repair of AAA or EVAR	Preoperative decision support
Kordzadeh et al. [16]	The role of artificial intelligence in the prediction of functional maturation of arteriovenous fistula	2019	266 patients who underwent radiocephalic arteriovenous fistula	Predictive model- Artificial neural network prediction model to forecast functional maturation of AV fistula	Preoperative decision support
Van Tongeren et al. [20]	Volume measurement for surveillance after EVAR using artificial intelligence	2025	98 CTA images of 49 patients analysed at day 30 and 1year post EVAR	Imaging model- PRAEVAorta 2- a software that detects aneurysm characteristics originally described in France	Post operative surveillance after EVAR
Van Veldhuizen et al. [17]	Machine learning based prediction of post operative infrarenal endograft apposition for AAA	2024	147 patients treated with standard EVAR	Predictive model- 3D Statistical shape modelling (SSM) – a software created to predict the shortest apposition length for endograft and determine suitability for EVAR	Preoperative decision support tool

Data Extraction

Data extraction was done by two independent authors and compiled on Microsoft Office Excel Version 16 software (Microsoft, Redmond, WA, USA). The information extracted from the selection studies focussed on the study characteristics (author, study title, year, country, dataset and split, baseline comparators and type of validation), the AI model designed and trained in the study, model performance metrics, the area of impact of the AI tool on operative planning and findings overview.

Synthesis

A meta-analysis was not feasible given the heterogeneity in study designs, datasets, participant characteristics, and outcome measures. Therefore, a qualitative narrative synthesis was undertaken. Data from each study were analysed according to the operative phase of AI implementation (preoperative, intraoperative, and postoperative) and the corresponding clinical domain of impact. Study characteristics and AI model details are summarised in Table 1, and the overall findings are presented in Table 2. Assessments of methodological quality and risk of bias using PROBAST and QUADAS-2 were incorporated into the interpretation and are displayed graphically in Figures 2-6. Visualisation plots for the QUADAS-2 assessments were generated using the robvis tool [21], while those for the PROBAST evaluations were created following the template proposed by Fernández-Félix et al. [22]. Across the included studies, AI was predominantly applied in the preoperative phase, with fewer studies addressing intraoperative and operative phases.

**Table 2:** Overview of results across included studies CTA: Computed tomography angiography, CT: Computed tomography, SSI: Surgical site infection, EVAR: Endovascular aneurysm repair, CEA: Carotid endarterectomy, CI: Confidence interval, ICC: Intraclass correlation coefficient, SAL: Shortest apposition length, sec: seconds, min: minutes. AUROC (Area Under the Receiver Operating Characteristic Curve) is a machine learning metric that quantifies a binary classification model's ability to distinguish between positive and negative classes across all possible classification thresholds. It is the area under the ROC curve, which plots the True Positive Rate (TPR) against the False Positive Rate (FPR). A perfect model has an AUROC of 1, a random model has an AUROC of 0.5, and a lower AUROC indicates a worse model. The Brier score ranges from 0 (perfect prediction) to 1 (worst prediction). Lower values indicate better performance. Brier scores below 0.25 generally indicate useful prediction; lower values reflect better overall accuracy. P value <0.05 suggest statistical significance.

Author	Data set and split	Baseline comparator	Validation type	Country	Model performance metrics (95% CI)	Summary of results
Caradu et al. [18]	n=100 CTA scans. Split not explicitly stated. 38 training CTA used, separate from the 100 CTA data set.	Manual segmentation of CTA by 2 vascular surgeons.	Internal (single centre observational retrospective)	France	Segmentation time: 90 sec vs 35 min. No CI reported. Concordance - Pearson’s coefficient correlation of > 0.90 (p < 0.0001)	Mean time for AI automatic segmentation of aortic lumen was 90secs per patient versus 35minutes for manual method with excellent concordance between both methods.
Chang et al. [13]	n=370 patients. 72,435 national cases from NSQUIP dataset -split 80% training, 20% validation. Fine tuning on separate data n=370 (study cohort).	Clinical judgement.	Internal (retrospective cohort)	United States of America	Predicted SSI reduction 29.2–45.3%; cost reduction15.0–29.7% No CI reported.	Objective risk-based assessment using predictive AI software to accurately match patients to ciNPT (closed incision negative pressure dressing) could reduce SSI rate by up to 45.3% and reduce cost per patient by up to 29.7%.
Li et al. [14]	n=166,369 CEA cases. 70% training and 30% validation.	Logistic regression	Multicentre internal (retrospective cohort)	Canada Saudi Arabia	XGBoost - (AUROC, 0.90; 95% CI, 0.89-0.91).	XGBoost, the best performing AI model in the study demonstrated good preoperative prediction of stroke or death at 1year post CEA with superior discriminative ability compared to traditional methods (AUROC 0.90 vs 0.58-0.74, 95%CI). Brier scores of 0.15, 0.14, and 0.11 for the preoperative, intraoperative and postoperative models on calibration plot indicating progressively better prediction of 1-year stroke or death after CEA.
Huang et al. [19]	n=36 20 training cohort. 16 validation cohort-9 with invasion, 7 without	Conventional preoperative CT assessment and intraoperative findings.	Hybrid. Retrospective training and prospective validation.	China	Sensitivity 88.9% (8/9; 95% CI 51.8–99.7%); Specificity 85.7% (6/7; 95% CI 42.1–99.6%). Operating time reduced (194 vs 309 min, p=0.007). Estimated blood loss reduced (400 vs 1150 mL, p<0.001).	The virtual vascular endoscope precisely identified inferior vena cava (IVC) tumour thrombus preoperatively in 8 out of 9 patients, confirmed by pathology. Median operating time and estimated blood loss were significantly lower with AI use (194mins vs 309mins, 400ml vs1150ml).
Kiessling et al. [15]	n=7912 80% training, 20% validation	Kaplan–Meier (KM) survival estimates	Internal (retrospective cohort)	Denmark	Brier score for KM at 10years - 0.2443 (95% CI 0.2407 -0.2488) Brier score for AI model at 10years- 0.206 (95% CI 0.1914 -0.2199).	AI model was more accurate than the gold standard Kaplan-Meier (KM) method, delivering better 10-year survival prediction. Brier score lower for AI across all time points; absolute improvement-3.8% at 10years (p<0.025).
Kordzadeh et al. [16]	n=266. 70% training, 15% evaluation and 15% validation.	Clinical assessment and duplex ultrasonography at 6weeks.	Prospective cohort (single centre)	United Kingdom	Accuracy >80% at optimal threshold p<0.01 (statistical significance is p<0.05) No CI reported.	In the study cohort, functional maturation (FM) of radio cephalic arteriovenous fistula (RCAVF) at 6weeks was preoperatively predicted by virtual AI model with >80% accuracy using only 10 patients’ variables.
Van Tongeren et al. [20]	N= 49 patients (98 CTA). Split not explicitly stated.	Validated semi-automated volumetry.	Multicentre internal (retrospective)	Netherlands United Kingdom	ICC 0.94 (95% CI 0.88–0.99); mean bias –0.01 cc	Notable correlation of AI versus semiautomated method with AI method enabling earlier detection of asymptomatic post EVAR complications. Consistent repeatability of AI method with mean bias negligible at -0.01cc
Van Veldhuizen et al. [17]	n=147 EVAR; dataset enriched with type-1a endo leaks. Split not explicitly stated.	Conventional anatomical assessment – CTA and duplex ultrasonography.	Internal (retrospective case-control)	Netherlands	Accuracy 77.6% for shortest apposition length No CI reported.	The AI model predicted shortest endograft apposition length with 77.6% accuracy using preoperative CTA, potentially reducing type 1a endo-leak risk from inadequate apposition. Sensitivity (correct classification of < 10 mm SAL) was 0.70 and specificity (correct classification of ≥ 10 mm SAL) was 0.79

Results

Summary of Search Results

The database search identified 817 records, and after removing duplicates, 568 unique studies remained. Title and abstract screening excluded 517 studies, leaving 51 articles for full-text assessment. Eight studies met the predefined eligibility criteria and were included in the final review (Figure 1).

Applications of AI in Operative Workflow

Preoperative: Seven of the eight studies in this review described the application of AI models in the preoperative stage of surgical workflow as outlined in Table 1, notably in decision support, risk prediction, and patient selection. These are the key components of preoperative surgical planning with the aim of mitigating morbidity and mortality.

Caradu et al. [18] assessed the quality and efficiency of a fully automated AI model in the detection of aortic lumen and delineating infrarenal abdominal aortic aneurysm (AAA) features, including thrombus presence, using the preoperative computed tomography angiography (CTA) of the study cohort. The conventional method used in the study involved manual segmentation of the CTA by two vascular surgeons (senior and junior). Segmentation provides information on AAA volume and morphology, which more accurately predict aneurysm growth rate and risk of rupture than the aneurysm diameter [23]. However, manual segmentation is time-consuming and has poor interobserver reproducibility. Detection of aneurysm evolution at the preoperative level is key to operative planning. The AI model segmentation time in this study ranged from 27 seconds to four minutes per patient compared to the five minutes to 80 minutes for the manual method. Correlation between the two methods was remarkable, with Pearson’s correlation coefficient of >0.90 (p < 0.0001). This performance translates to relatively efficient treatment decisions, prognosis assessment, and human error reduction, and allows for high-volume CTA analysis, improving operative workflow from the outset.

Chang et al. [13] applied a deep learning prediction model to patients planned for infrainguinal vascular surgery to identify high-risk patients and preoperatively mitigate surgical site infection (SSI) in this group. SSI is common following infrainguinal vascular surgery, resulting in prolonged hospital stay, readmissions, sepsis, return to theatre, high cost, and significant morbidity, including graft loss and major amputation. Closed incision negative pressure therapy (ciNPT) has been shown in literature to reduce postsurgical complications in high-risk patients [24-28]. The AI model in this study was developed to assist in predicting the patient-specific risk of postoperative SSI at the preoperative stage. ciNPT are expensive, and subjective clinical judgement could lead to overuse in low-risk groups or underuse in high-risk groups, hence the need for accurate patient selection. Chang et al. [13] reported that ciNPT reduced SSI rates in high-risk vascular surgery patients (6.8% vs 20.9%). Also, an objective AI model risk assessment to guide ciNPT use could potentially reduce SSI incidence by 29.2%-45.3% and costs per patient by 15.0%-29.7%.

Li et al. [14] trained six machine learning model to predict one-year stroke or death outcome post-carotid endarterectomy (CEA) operation. CEA is a stroke prevention procedure with significant risk, the risk profile influenced by patient demographics (older age group), comorbidities (for example heart failure, diabetes etc) and unfavourable anatomical characteristics (contralateral carotid stenosis). Risk stratification and preoperative prediction of outcome are key for CEA, with the study identifying XGBoost as the best performing AI model at the preoperative stage, achieving an area under the receiver operating characteristic curve (AUROC) of 0.90; 95% confidence interval (CI) 0.89-0.91. In comparison to logistic regressor or Cox mode (conventional statistical method of predicting outcome) with AUROC 0.58 to 0.74, this prognostic AI model demonstrates superior discrimination that guides clinical decision making. Following the impressive performance, additional XGBoost models were built in for the intraoperative and postoperative stages of CEA.

High-risk patients, identified at the preoperative phase, are either referred for further optimisation prior to surgery or solely for medical management. The clinical impact of this AI model lies in its ability to inform CEA decision making by risk stratification, enabling tailored multi-level strategies across the preoperative, intraoperative and postoperative phases. This means a patient identified as high risk at any operative stage may benefit from either optimisation, alternative management, intensive care monitoring, early follow-up plan or a combination of these interventions.

Huang et al. [19] discussed the use of an AI-driven virtual vascular endoscope in 36 patients with renal tumours and inferior vena cava (IVC) thrombi undergoing robotic-assisted IVC thrombectomy (RA-IVCT). RA-IVCT is inherently a multidisciplinary procedure, typically involving urology and or general surgery with vascular surgery involvement or on standby [29]. The model reconstructed preoperative CT into virtual reality images to preoperatively predict IVC wall invasion, a crucial determinant of surgical strategy and prognosis. In the validation cohort, the model recorded high accuracy (8/9 with invasion, 6/7 without) confirmed by postoperative histology, demonstrating 88.9% sensitivity and 85.7% specificity with one false positive and one false negative identified. The validation cohort had significantly shorter median operative time and lower blood loss than the training cohort (194 vs 309 min, p=0.007; 400 vs 1150 mL, p<0.001). p value <0.05 suggest statistical significance.

Van Veldhuizen et al. [17] studied the use of an AI model to predict the postoperative infrarenal endograft apposition length following standard EVAR for AAA using preoperative CTA. Unfavourable anatomical variation in infrarenal neck features is associated with increased risk of type 1a endoleak after EVAR [30-32]. The AI model analysed the complex three-dimensional morphological variation of the aortic neck, evaluating the suitability of EVAR for a patient based on the preoperative aortic geometry on CTA. It assessed long-term durability while determining if other options such as like fenestrated EVAR (FEVAR) or open surgery may be more appropriate. The study reported 77.6% accuracy by the AI model in prediction of shortest endograft apposition length based on the pre-operative scan, highlighting its potential as a decision support tool. However, enrichment of the dataset with 1a endoleak cases may have introduced bias towards improved AI performance and should be considered when interpreting results.

Kiessling et al. [15] studied the novel use of an AI model to estimate patient survival after elective AAA repair by either open surgical repair (OSR) or EVAR over various time points up to 10 years excluding 23 patients who had re-operations. The retrospective analysis was done using predefined data on patient demographics, comorbidities, and aneurysm characteristics and operative details - type of repair (OSR or EVAR). Outcomes recorded were survival status and follow-up duration. The AI model’s predictive performance was evaluated using the Brier score [33] and benchmarked against the Kaplan-Meier method [34], a validated widely applied conventional statistical approach for survival analysis in medical research. At all assessed timelines (90 days, one, three, five, seven, 10 years), significantly lower Brier scores were achieved by AI, indicating that its predicted survival probabilities aligned more closely with the observed outcomes compared to conventional method. Overall, the study reported that the AI model provided enhanced predictive accuracy, with a 3.8% absolute improvement at 10 years p<0.025 (p<0.05 suggests statistical significance). In view of the long-term survival differences between open repair and EVAR especially in young patients [35,36], an accurate predictive model could significantly impact preoperative choice of surgical approach.

Kordzadeh et al. [16] demonstrated the use of AI model to predict functional maturation (FM) of radiocephalic arteriovenous fistula RCAVF, the first-line choice of vascular access in haemodialysis patients. RCAVF maintain patency for years with minimum intervention once functionally mature. FM varies widely across centres (40-70%) and is impacted by several factors [37,38]. Outcome at six weeks showed that the AI model preoperatively predicted FM with >80% accuracy using only 10 patients’ variables. Statistically significant discriminatory performance was demonstrated by the model at optimal sensitivity-specificity threshold (p<0.01). These result shows the potential of the AI model as a replicable, adaptable clinical decision support tool with reduced heterogeneity across centres.

Intraoperative: Li et al. [14] retrospective study demonstrated AI model enabled real-time intraoperative risk updates during CEA, functioning as a perioperative decision-support tool. Although the model was expanded to include intra- and postoperative variables later, predictive performance (AUROC of 0.90; 95%CI 0.89-0.91) remained unchanged with intraoperative variables with risk stratification driven primarily by preoperative characteristics.

Postoperative: Van Tongeren et al. [20] evaluated an AI model for post-EVAR surveillance, like prior work by Caradu et al. [18]. Volumetric assessment is a more sensitive indicator of aneurysm sac growth, regression, or stability than diameter, but its clinical utility has been limited by the labour-intensive nature of conventional methods [39,40]. The AI model automated this process, showing comparable agreement with a validated semi-automated technique (Intraclass Correlation Coefficient (ICC) 0.94, 95%CI 0.88-0.99) and near-perfect reproducibility with negligible bias (-0.01 cc). The ICC is a value between 0 and 1 that measures the consistency or agreement of measurements made by two or more raters, or in repeated measurements by the same rater. Within the standard 30-day and one-year surveillance framework, AI-driven volumetric analysis may facilitate earlier detection of asymptomatic complications post-EVAR, streamline operative decision-making, and enable timely re-intervention, thereby potentially reducing aneurysm-related mortality. However, the exclusion of 15 out of 64 cases in the study following segmentation failures from poor CTA quality could potentially inflate AI performance over conventional methods, additionally AI software measuring a longer aortic segment may affect measurement comparability, highlighting the need for external validation.

Quality and Risk of Bias Summary

The methodological quality of the included studies was relatively high, with most studies demonstrating low risk of bias across all assessed domains as shown in Figures 2-6. PROBAST was applied to predictive modelling studies, while QUADAS-2 was used for diagnostic imaging studies [2,12]. Key exceptions were Chang et al. [13], where study cohort was followed prospectively to 90 days, however NSQIP (National Surgical Quality Improvement Program) defines SSI within 30 days. This outcome window discord introduced unclear risk of bias concerns in the outcome, analysis and applicability concerns. Furthermore, data set enrichment with type 1 endoleak cases in Van Veldhuizen et al. [17] study introduced high risk of bias driven by participants with unclear analysis and applicability concerns. In Van Tongeren study [20], patient selection demonstrated a high risk of bias, as 15 of 64 cases (23%) were excluded post-analysis due to segmentation failures and non-detection of the infrarenal aortic zone from suboptimal CTA quality, thus introducing some concerns in flow and timing shown in Figure 5 and Figure 6. Additionally, the subtle difference in the reference standard, also presented moderate risk and applicability concerns shown in Figure 6. The reference standard for semiautomated validated method measured aneurysm volume from 10 mm below the renal arteries to 10 mm above the aortic bifurcation while AI software measured directly from the renal arteries to the bifurcation. However, bifurcation was consistently defined as the point of full separation of the common iliac arteries.

Overall Impact of AI on Workflow

Caradu et al. [18] and Van Tongeren et al. [20] studies showed AI use improved operative workflow efficiency by automating manually demanding imaging segmentation and volumetric analysis processes. The role of AI in enhancing decision making through advanced risk stratification, outcome prediction and anatomical suitability assessments is demonstrated in the studies by Chang et al., Li et al., Van Veldhuizen et al. and Kiessling et al. [13-15,17] shown in Table 2.

Furthermore, several studies demonstrate promising AI potential to optimise patient outcomes by enabling tailored preoperative management (Chang et al., Li et al., Kordzadeh et al.) reducing postoperative complications (Chang et al., Van Veldhuizen et al.) and supporting intraoperative strategies (Huang et al., Li et al.) [13,14,16,17,19]. The reproducibility of AI models shown in studies by Caradu et al., Van Tongeren and Kordzadeh et al. demonstrated the feasibility of standardised care pathway by minimising interobserver variability and outcome heterogeneity [16,18,20].

Opinion on Safety

The included studies reported moderately favourable safety outcomes with AI application. Caradu et al. [18] reported that automated AAA segmentation achieved excellent concordance with manual methods p<0.0001 (p<0.05), while Van Tongeren et al. [20] showed strong agreement between AI volumetric EVAR surveillance and semi-automated techniques (ICC 0.94). Chang et al. [13] found that risk-based stratification for ciNPT reduced SSI rates 6.8% vs 20.9%, p<0.001 (p<0.0.5), and Li et al. [14] demonstrated superior prognostic accuracy of XGBoost after CEA (AUROC 0.90 vs 0.58-0.74).

In operative planning, Huang et al. [19] achieved accurate detection of IVC invasion (sensitivity 88.9%, specificity 85.7%) with reduced operative time and blood loss, while Van Veldhuizen et al. [17] predicted endograft apposition with 77.6% accuracy, lowering risk of type 1a endoleak. Kiessling et al. [15] showed improved survival prediction over the conventional Kaplan-Meier method (+3.8% at 10 years, p<0.025), and Kordzadeh et al. [16] demonstrated AI-based AV fistula maturation prediction with >80% accuracy. Overall, these studies indicate that AI enhances surgical safety through accurate prediction, risk reduction, and complication prevention across the perioperative pathway.

Discussion

The emerging role of AI in the operative workflow within vascular surgery is highlighted in this review. Seven studies demonstrated AI value in preoperative planning, one study reported intraoperative decision support, and two studies showed AI model guiding postoperative monitoring and complications. The principal applications involved risk prediction, outcome forecasting, automated anatomical segmentation and complication prevention with the AI models generally outperforming conventional manual techniques or statistical methods as detailed in Table 2, though evidence remains preliminary.

Key Findings and Comparison With Existing Literature

Compared to other non-cardiac surgery, patients undergoing major vascular surgery have higher incidence of adverse outcomes [41,42]. This peculiar landscape of vascular surgery demands effective preoperative planning, accurate personalised risk prediction [42] and explains why majority of the studies in this review have focussed on AI model application at this phase. Automated imaging tools for example PRAEVAorta [18,20] and predictive algorithms including XGBoost for carotid endarterectomy [14] and neural multi-task logistic regression for abdominal aortic aneurysm survival [15] demonstrated notable improvements in discrimination and reproducibility compared with traditional methods. These findings are consistent with literature from other surgical domains, where AI has shown predominance in preoperative and diagnostic functions [43,44].

Impact on Patient Safety

AI-driven risk stratification demonstrated lower surgical site infection rates [13], improved patient selection for endovascular repair [17], and better prediction of fistula maturation [16]. However, these advantages are largely inferred from model accuracy rather than clinical impact trials. Huang et al. [19] showed that virtual vascular endoscopy reduced operative time and blood loss during IVC thrombectomy, but evidence remains limited to small, single-centre studies. Overall, the findings, while promising, mainly reflect improvements in workflow and procedural precision rather than validated improvements in patient outcomes limiting the ability to draw definite conclusions on patient safety.

Clinical Implications

AI offers considerable advantages in efficiency, standardisation and replicability compared to conventional approaches. Automation of labour-intensive processes as seen in CT segmentation and volumetric measurement substantially reduced analysis time while maintaining considerable accuracy [18,20]. Predictive modelling in this review demonstrated enhanced preoperative decision-making, supporting more tailored management strategies. However, AI in its current form should be considered as a workflow adjunct complementing not replacing clinical judgement. In line with evolving standards for transparency in artificial intelligence research, adherence to EQUATOR Network extensions for AI, including TRIPOD-AI and CONSORT-AI, will be essential to improve methodological rigor, reproducibility, and reporting consistency across future studies. Furthermore, as healthcare AI systems are increasingly recognised as high-risk technologies under emerging frameworks such as the European Union Artificial Intelligence Act, alignment with these standards will be critical to ensure the safe and ethical integration of AI tools into vascular surgical practice [2,10].

Limitations and Ethical Concerns

Despite the promising potential of AI application in vascular operative workflow, several limitations constrain both the included studies and the review. The small number of included studies (eight) in this review limits the interpretability of the findings. Most studies were retrospective, single-center with small and heterogenous endpoints [13] which limited the feasibility of conducting a meta-analysis. The scarce external validation [15,18,19], absence of bias analysis, poor reporting of decision curve and calibration analyses limits the clinical utility of these models.

The efficiency of the AI model relies on the image quality of the CT (for example PRAEVaorta) [18,20] and notably patient training data [45], however none of the studies assessed algorithm bias, which is a known challenge with AI models. This is a research gap which if addressed could evaluate AI performance across diverse patient groups to ensure equitable AI adoption in vascular surgery.

Ethical considerations with AI use including data governance, privacy and accountability highlight the need for stringent measures that prevent data theft, exploitation or misuse [46] and must align with evolving regulatory frameworks for example the EU (European Union) Artificial Intelligence Act [10] amongst others. Additionally, AI reporting standards (for example TRIPOD-AI, CONSORT-AI) should be adopted to improve transparency, methodological rigor and reproducibility [2].

Future Research

Robotic-assisted vascular surgery was not examined in detail given the limited available evidence, though early reports suggest feasibility and warrant further research. Further research could address algorithm bias ensuring equitable treatment across all patient groups, prospective multicentre validation, calibration and decision-curve analysis to assess clinical impact and clinical trial integration to evaluate safety, cost-effectiveness and workflow impact.

## Conclusions

AI applications in vascular surgery are most advanced in the preoperative phase, focusing on imaging automation and risk prediction, with fewer studies addressing intraoperative or postoperative use. Overall, AI models achieved good technical performance for example AUROC 0.90 for CEA outcomes, ICC 0.94 for EVAR volumetry >80% accuracy for AVF maturation, indicating potential to enhance workflow efficiency and decision support. However, current evidence is limited by small, retrospective, and heterogeneous studies lacking prospective external validation and algorithm bias assessment. The promising performance of AI models over conventional method should be interpreted with appropriate caution.

Future research should focus on robotic assisted vascular surgery, prospective, multicentre, transparently reported validation studies, incorporating fairness across diverse patient groups, calibration, and regulatory alignment. AI holds significant promise in revolutionizing vascular care by enabling personalized treatment plans, improving procedural outcomes, and optimizing system-level efficiency. However, broader adoption will require ongoing interdisciplinary collaboration, robust data governance, and ethical oversight to ensure that AI-driven solutions are both effective and equitable in clinical practice. As AI integration in vascular surgery continues to emerge, with ongoing evaluations in vascular diagnostics, perioperative medicine, risk stratification and outcome assessment, it remains a promising adjunct in improving patient outcomes, operative workflow and safety.

## Appendices

Appendix 1

**Table 3:** Preferred Reporting Items for Systematic reviews and Meta-Analyses (PRISMA) 2020 checklist.

Section and Topic	Item #	Checklist item	Location where item is reported
TITLE			
Title	1	Identify the report as a systematic review.	Title
ABSTRACT			
Abstract	2	See the PRISMA 2020 for Abstracts checklist.	Abstract
INTRODUCTION			
Rationale	3	Describe the rationale for the review in the context of existing knowledge.	Introduction and Background
Objectives	4	Provide an explicit statement of the objective(s) or question(s) the review addresses.	Introduction and Background
METHODS			
Eligibility criteria	5	Specify the inclusion and exclusion criteria for the review and how studies were grouped for the syntheses.	Inclusion and exclusion criteria
Information sources	6	Specify all databases, registers, websites, organisations, reference lists and other sources searched or consulted to identify studies. Specify the date when each source was last searched or consulted.	Search strategy
Search strategy	7	Present the full search strategies for all databases, registers and websites, including any filters and limits used.	Search strategy
Selection process	8	Specify the methods used to decide whether a study met the inclusion criteria of the review, including how many reviewers screened each record and each report retrieved, whether they worked independently, and if applicable, details of automation tools used in the process.	Selection and screening
Data collection process	9	Specify the methods used to collect data from reports, including how many reviewers collected data from each report, whether they worked independently, any processes for obtaining or confirming data from study investigators, and if applicable, details of automation tools used in the process.	Data extraction
Data items	10a	List and define all outcomes for which data were sought. Specify whether all results that were compatible with each outcome domain in each study were sought (e.g. for all measures, time points, analyses), and if not, the methods used to decide which results to collect.	Data extraction. Results; Table 2
	10b	List and define all other variables for which data were sought (e.g. participant and intervention characteristics, funding sources). Describe any assumptions made about any missing or unclear information.	Data extraction. Results; Table 1
Study risk of bias assessment	11	Specify the methods used to assess risk of bias in the included studies, including details of the tool(s) used, how many reviewers assessed each study and whether they worked independently, and if applicable, details of automation tools used in the process.	Quality and risk of bias assessment
Effect measures	12	Specify for each outcome the effect measure(s) (e.g. risk ratio, mean difference) used in the synthesis or presentation of results.	Not applicable. No meta-analysis
Synthesis methods	13a	Describe the processes used to decide which studies were eligible for each synthesis (e.g. tabulating the study intervention characteristics and comparing against the planned groups for each synthesis (item #5)).	Synthesis
	13b	Describe any methods required to prepare the data for presentation or synthesis, such as handling of missing summary statistics, or data conversions.	Synthesis
	13c	Describe any methods used to tabulate or visually display results of individual studies and syntheses.	Synthesis. Table 1-2
	13d	Describe any methods used to synthesize results and provide a rationale for the choice(s). If meta-analysis was performed, describe the model(s), method(s) to identify the presence and extent of statistical heterogeneity, and software package(s) used.	Not applicable
	13e	Describe any methods used to explore possible causes of heterogeneity among study results (e.g. subgroup analysis, meta-regression).	Not applicable
	13f	Describe any sensitivity analyses conducted to assess robustness of the synthesized results.	Not applicable
Reporting bias assessment	14	Describe any methods used to assess risk of bias due to missing results in a synthesis (arising from reporting biases).	Not applicable. Quantitative pooling not done.
Certainty assessment	15	Describe any methods used to assess certainty (or confidence) in the body of evidence for an outcome.	Certainty of evidence not formally assessed.
RESULTS			
Study selection	16a	Describe the results of the search and selection process, from the number of records identified in the search to the number of studies included in the review, ideally using a flow diagram.	Results. Fig 1
	16b	Cite studies that might appear to meet the inclusion criteria, but which were excluded, and explain why they were excluded.	Not included
Study characteristics	17	Cite each included study and present its characteristics.	Results. Table 1
Risk of bias in studies	18	Present assessments of risk of bias for each included study.	ResultsàQuality and risk of bias summary. Fig 2-6
Results of individual studies	19	For all outcomes, present, for each study: (a) summary statistics for each group (where appropriate) and (b) an effect estimate and its precision (e.g. confidence/credible interval), ideally using structured tables or plots.	ResultsàTable 2
Results of syntheses	20a	For each synthesis, briefly summarise the characteristics and risk of bias among contributing studies.	Results
	20b	Present results of all statistical syntheses conducted. If meta-analysis was done, present for each the summary estimate and its precision (e.g. confidence/credible interval) and measures of statistical heterogeneity. If comparing groups, describe the direction of the effect.	Not applicable
	20c	Present results of all investigations of possible causes of heterogeneity among study results.	Not applicable
	20d	Present results of all sensitivity analyses conducted to assess the robustness of the synthesized results.	Not applicable
Reporting biases	21	Present assessments of risk of bias due to missing results (arising from reporting biases) for each synthesis assessed.	Not applicable
Certainty of evidence	22	Present assessments of certainty (or confidence) in the body of evidence for each outcome assessed.	Not applicable
DISCUSSION			
Discussion	23a	Provide a general interpretation of the results in the context of other evidence.	Discussion
	23b	Discuss any limitations of the evidence included in the review.	Limitation and ethical concern
	23c	Discuss any limitations of the review processes used.	Limitation and ethical concern
	23d	Discuss implications of the results for practice, policy, and future research.	Clinical implications. Future research.
OTHER INFORMATION			
Registration and protocol	24a	Provide registration information for the review, including register name and registration number, or state that the review was not registered.	Methods
	24b	Indicate where the review protocol can be accessed, or state that a protocol was not prepared.	Not applicable
	24c	Describe and explain any amendments to information provided at registration or in the protocol.	Not applicable
Support	25	Describe sources of financial or non-financial support for the review, and the role of the funders or sponsors in the review.	Not applicable
Competing interests	26	Declare any competing interests of review authors.	Disclosures
Availability of data, code and other materials	27	Report which of the following are publicly available and where they can be found: template data collection forms; data extracted from included studies; data used for all analyses; analytic code; any other materials used in the review.	Not applicable
